# Anti‐tumor necrosis factor‐α monotherapy versus combo therapy with immunosuppressant in pediatric inflammatory bowel disease: A real‐life study

**DOI:** 10.1002/jpn3.70280

**Published:** 2025-11-20

**Authors:** Flora Fedele, Massimo Martinelli, Caterina Strisciuglio, Elena Scarpato, Annamaria Staiano, Erasmo Miele

**Affiliations:** ^1^ Department of Translational Medical Science, Section of Pediatrics University of Naples “Federico II” Naples Italy; ^2^ Department of Woman, Child and General and Specialistic Surgery University of Campania “Luigi Vanvitelli” Napoli Italy

**Keywords:** anti‐TNF therapy, children, immunosuppressive therapy, therapeutic drug monitoring

## Abstract

**Objectives:**

The aim of the study is to evaluate the efficacy of anti‐tumor necrosis factor (TNF)‐α monotherapy versus combination anti‐TNF‐α and immunosuppressive therapy.

**Methods:**

A single‐center, retrospective, observational study was conducted on inflammatory bowel disease (IBD) children. Patients with at least 6 months of follow‐up were enrolled and divided into two groups based on therapy. Combo group included children on combination anti‐TNF‐α and immunosuppressant therapy; children undergoing anti‐TNF‐α monotherapy were assigned to Mono group.

**Results:**

One hundred and seventeen children were enrolled, of whom 74 (63.2%) were affected by Crohn's disease (CD) and 43 (36.8%) by ulcerative Colitis (UC) (median age at diagnosis: 11.6 years; range 2.1–16.9; M/F: 56/61). Eighty patients (68.4%) were included in combo group and 37 (31.6%) in mono group. The median follow‐up was 2.6 years (0.5–11.3). Twenty‐three patients out of 80 (28.7%) in Group 1 showed therapy failure compared with 21/37 (56.8%) children in Mono group (*p* = 0.04). CD patients in monotherapy showed a significantly increased risk of therapy failure than those treated with combination therapy (*p* < 0.001). Conversely, no difference was found in UC children (*p* = 0.7). Children undergoing a reactive approach showed more frequent therapy failure compared to proactive in both groups (combo group: 41.7% vs. 4.3%; *p* = 0.01; mono group: 87.5% vs. 20%; *p* = 0.01). In a multivariate regression model, the use of a proactive approach and combination therapy was independently associated with anti‐TNF‐α durability (odds ratio [OR] = 22.1, OR = 12.9).

**Conclusion:**

Combination therapy reduced overall anti‐TNF‐α failure in CD children, but not in UC patients. Additionally, a proactive approach was associated with increased anti‐TNF‐α durability.

## Abstract (highlights)

**What is Known:**

- The use of anti‐tumor necrosis factor (TNF)‐α biological drugs for the treatment of pediatric inflammatory bowel disease (IBD) is increased, but some patients show loss of response to anti‐TNF‐α inhibitors.
- The results of studies about combined immunosuppressive and anti‐TNF‐α therapy and anti‐TNF‐α monotherapy in pediatric IBD are conflicting.

**What is New:**

- Combination therapy and proactive approach were factors independently associated with increased anti‐TNF‐α durability.
- The use of combination therapy and proactive therapeutic drug monitoring should be considered to improve clinical and laboratory outcomes in IBD children.

## Graphical abstract

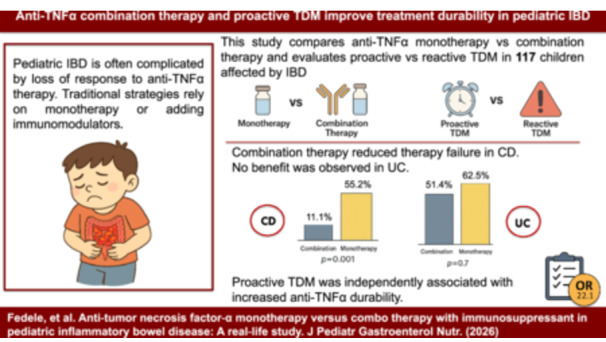

## 1 INTRODUCTION

During the last decade, the use of anti‐tumor necrosis factor (TNF)‐α biological drugs for the treatment of pediatric Crohn's disease (CD) and ulcerative colitis (UC) exponentially increased. Despite their efficacy, a substantial number of patients with inflammatory bowel disease (IBD) do not respond or lose response to anti‐TNF‐α inhibitors. This may be related to suboptimal drug concentrations and/or anti‐drug antibodies production.^1^, ^2^ Indeed, high levels of anti‐TNF‐α reduce the formation of anti‐drug antibodies, which may be responsible of secondary loss of response (LOR). Secondary LOR is described as a clinical failure during the maintenance phase after an initial response to induction therapy.^3^ Currently, anti‐TNF‐α antibodies formation can be reduced through two therapeutic strategies: combination (combo) therapy with conventional immunosuppressants (IM) or anti‐TNF‐α monotherapy (mono) with proactive drug level monitoring.^4^ The current evidence is still inconclusive toward the use of one therapeutic strategy over another.^5^, ^6^ In adult patients, recent systematic reviews and meta‐analysis showed that combo therapy decreased antibodies formation^7^ and had slight benefits in inducing clinical remission in active CD patients compared to mono.^8^ Regarding therapeutic drug monitoring (TDM), the TAXIT^9^ and the TAILORIX,^10^ two randomized controlled trials (RCTs) conducted on adult patients, failed to demonstrate the superiority of level‐based over clinically based adjustment of infliximab (IFX) treatment. In contrast, the PAILOT study demonstrated that proactive approach was superior to reactive in children with CD treated with adalimumab (ADA).^11^ However, data on the role of combo therapy in pediatric IBD are still insufficient and conflicting.^12^, ^13^, ^14^

The primary aim of the present study was to compare the efficacy of anti‐TNF‐α mono versus combo therapy in children with IBD. The secondary aim was to evaluate the two therapeutic strategies in terms of anti‐TNF‐α immunogenicity, pharmacokinetics, and safety.

## 2 METHODS

### 2.1 Ethics statement

The study was approved by the Institutional Review Board (IRB) of the University of Naples “Federico II” with the protocol registration number 212/21. The study complies with the Declaration of Helsinki on research ethics. Informed consent was obtained from all parents and from children, when appropriate.

### 2.2 Study design and population

This was a retrospective, observational, single‐center study conducted on children followed at the pediatric IBD regional referral center of the Department of Translational Medical Science, Section of Pediatrics, University of Naples “Federico II.” All children with CD or UC receiving treatment with anti‐TNF‐α between June 2006 and February 2023 were identified. The inclusion criteria were: diagnosis of IBD according to the Porto Criteria^15^; age at diagnosis of 18 years or younger; follow‐up of at least 6 months. On the basis of the therapeutic strategies, we retrospectively identified two groups of patients: the combo group and the mono group. Enrolled children were included in the combo group if the IM was introduced before anti‐TNF‐α therapy or during the induction phase and continued concomitantly with the anti‐TNF‐α drug. The therapeutic strategies were adopted according to the evolving ESPGHAN guidelines.^5^, ^6^, ^16^, ^17^ Briefly, in children affected by CD, anti‐TNF‐α therapy was mainly started with a top‐down strategy in case of perianal disease or in case of complicated phenotype, while in the remaining cases it was considered in case of active disease despite IM monotherapy.^5^, ^16^ In the setting of UC, anti‐TNF‐α was started as a rescue therapy in those patients experiencing acute severe colitis (ASC), who were unresponsive to first‐line steroid therapy, or in case of chronic active colitis resistant to conventional IM.^6^, ^17^ With regard to TDM until 2020 patients of our cohort were followed with a reactive approach, with dose adjustments of anti‐TNF‐α made only following disease relapse. Subsequently, a proactive approach was more frequently used, based on the latest guidelines for the management of pediatric CD^5^; this approach consists of periodic monitoring level drug and intensification of dose based on trough levels (TL), regardless of disease activity. ADA, IFX, antibodies to ADA (ATA), and antibodies to IFX (ATI) serum levels were assayed in samples collected just before the administration of the subsequent dose. Triplicate measurements were performed for each sample. A commercial and validated enzyme‐linked immunosorbent assay was used for the monitoring of drugs and anti‐drug antibodies according to the manufacturer's instructions (R‐BioPharm, Melegnano, Italy). The detection limit for ADA and IFX was 0.1 mcg/mL, whereas for ATAs and ATIs it was 0.06 ng/mL. Specifically, the first proactive TDM in ADA‐treated patients was performed just before the third injection, while in IFX‐treated patients just before the fourth infusion. Subsequent monitoring in our patients was performed before each infusion/injection. In the proactive group anti‐TNF‐α TL were optimized to reach the currently required thresholds: ≥5 μg/mL for IFX and ≥7.5 μg/mL for ADA.^5^, ^6^

The following data were collected at diagnosis, at the introduction of the biological drug and at 6, 12, 18, 24, and 36 months of follow up: demographic data; disease activity based on pediatric ulcerative colitis activity index (PUCAI) and pediatric crohn disease activity index (PCDAI); laboratory data including anti‐TNF TL and antibodies, when available; disease location at diagnosis based on the Paris Classification^18^; medical therapy. In addition, primary nonresponse (PNR), or secondary LOR, reactive or proactive biologic optimization, requirement for escalation of anti‐TNF‐α dosing or frequency, the presence of side effects to therapy and the need for surgical treatment were recorded during the follow‐up.

### 2.3 Outcomes measures

The primary outcomes of the study were the cumulative probability of overall failure and secondary LOR to anti‐TNF‐α in children in the two treatment groups. Failure was defined as a therapy switch due to PNR or secondary LOR or the appearance of side effects. Secondary LOR was defined as the occurrence of clinical and/or laboratory relapse, leading to the addition of an immunomodulatory agent in patients belonging to the mono group, or to anti‐TNF‐α withdrawal in both groups.^19^, ^20^ Secondary outcomes included: PNR; difference in the occurrence of side effects; percentage of clinical remission at 12, 24, and 36 months; differences in median TL; percentage of colectomy in children affected by UC. PNR was defined as failure to achieve clinical response (described as a decrease in PCDAI < 12.5 point or in PUCAI < 20 points) after the completion of the induction phase of anti‐TNF‐α therapy, defined as just before the third injection for ADA and just before the fourth infusion for IFX. Clinical remission was defined by PUCAI or PCDAI ≤ 10.

### 2.4 Statistical analysis

Variables were screened for their distribution, and appropriate parametric or non‐parametric tests were adopted as necessary. The Mann–Whitney test for continuous variables and the *χ* ^2^ and Fisher's exact tests for categorical variables were used. The Kaplan–Meier method was used to estimate the cumulative risk of anti‐TNF‐α failure and secondary LOR during the follow‐up. Differences between curves were tested using the Log‐rank test. Multivariate conditional logistic regression analysis was used to explore the odds associated with drug durability. Drug durability was defined as the number of children continuing anti‐TNF‐α at the end of the follow‐up. Only the variables which resulted significantly associated at the univariate analysis were included in the model (*disease type, combo therapy, type of biologic and proactive drug monitoring*). Statistical significance was predetermined as *p* < 0.05. SPSS version 28 was used for all statistical analyses.

## 3 RESULTS

### 3.1 Baseline characteristics at diagnosis

A total of 131 patients affected by IBD undergoing anti‐TNF‐α therapy were identified. Fourteen children were excluded. Figure S1 shows the subjects' progression through the study. One hundred and seventeen children (median age at diagnosis: 11.6 years; range 2.1–16.9; M/F: 56/61) were finally included in the study, of whom 74 (63.2%) patients were affected by CD and 43 (36.8%) by UC. Demographic, clinical, and laboratory data at diagnosis are reported in Table S1.

### 3.2 Clinical characteristics at the time of enrollment

Enrolled patients started anti‐TNF‐α at a median age of 13.1 years (2.9–17.5) and with a median time of 13 months (0–142) from diagnosis. One‐hundred‐ten out of 117 (94%) started anti‐TNF‐α therapy from 2013. Eighty patients (68.4%) practiced IM in association to anti‐TNF‐α and were included in the combo group (Group 1), while 37 (31.6%) children underwent monotherapy with anti‐TNF‐α (Group 2). The anti‐TNF‐α drugs used were: IFX (combo group: 59 [73.8%]; mono group: 25 [67.6%], *p* = 0.5) and ADA (combo group: 21 [26.3%]; mono group: 12 [32.4%]). In combo group, the IM used were methotrexate (MTX) in 61 (76.3%) children and azathioprine in the remaining 19 (23.8%) children. Clinical characteristics at the time of anti‐TNF‐α therapy starting are reported in Table 1. The median induction dose in UC patients was significantly higher than in CD patients (6.4 [4–12] vs. 5 [5–7.9]; *p* = 0.004). Most UC children (65.1%) were presenting ASC flare at anti‐TNF‐α starting.

**Table 1:** Clinical characteristics at T0 of the enrolled children.

Characteristics	Combo group (*n* = 80)	Mono group (*n* = 37)	*p*
Median age, years (range)	13.2 (4–16.7)	13.2 (2.9–17.5)	0.9
Median time from diagnosis, months (range)	17 (0–142)	6 (0–45)	0.003
CD (*n*, %)	45 (56.3)	29 (78.4)	0.02
Median PCDAI	11.2 (0–50)	10 (2.5–47.5)	0.4
Paris classification (*n*, %)			
Ileum only (L1)	10 (27.8)	1 (9.1)	0.4
Colon only (L2)	10 (27.)	0	0.1
Ileum and colon (L3)	16 (44.4)	10 (90.1)	0.02
UC (*n*, %)	35 (43.8)	8 (21.6)	0.02
Median PUCAI	50 (5–85)	57.5 (5–70)	0.9
Paris classification (*n*, %)			
Ulcerative proctitis (E1)	1 (4)	0	1
Left‐sided colitis (E2)	—	—	
Extensive colitis (E3)	5 (20)	1 (16.7)	1
Pancolitis (E4)	19 (76)	5 (83.3)	1
Immunosuppressant			NA
Methotrexate	61 (76.3)	—	
Azathioprine	19 (23.8)	—	
Biologics (*n*, %)			
IFX	59 (73.8)	25 (67.6)	0.5
Dosage, median (range)	5.9 (5–12)	6 (5–10)	0.8
5 mg/kg	30 (53.6)	12 (60)	0.8
>5 mg/kg	26 (46.4)	8 (40)	
10 mg/kg	10 (17.9)	2 (10)	0.5
ADA	21 (23.6)	12 (32.4)	0.5
Dosage pro/kg, median (range)	1.01 (0.7–1.3)	1.3 (0.9–1.6)	1

Abbreviations: ADA, adalimumab; CD, Crohn's disease; IFX, infliximab; PUCAI, pediatric ulcerative colitis activity index; UC, ulcerative colitis.

### 3.3 Data at the follow‐up

#### 3.3.1 Primary outcomes

**Figure 1:**
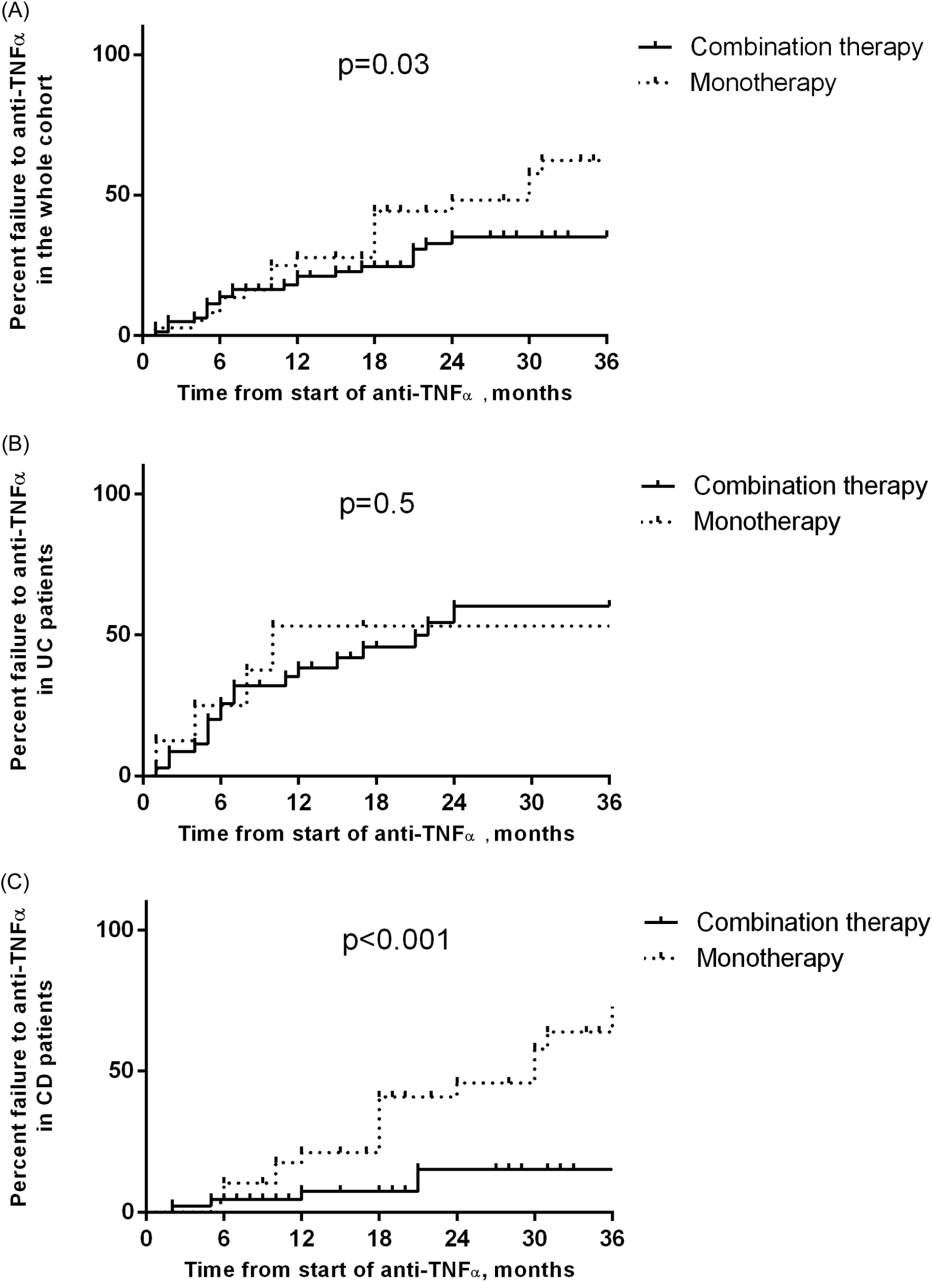
Overall failure to anti TNF‐α agents in pediatric IBD patients treated with combo therapy or monotherapy: (A) Cumulative risk in the whole cohort; (B) Cumulative risk in UC patients; (C) Cumulative risk in CD patients. CD, Crohn's disease; IBD, inflammatory bowel disease; TNF, tumor necrosis factor; UC, ulcerative colitis.

**Figure 2:**
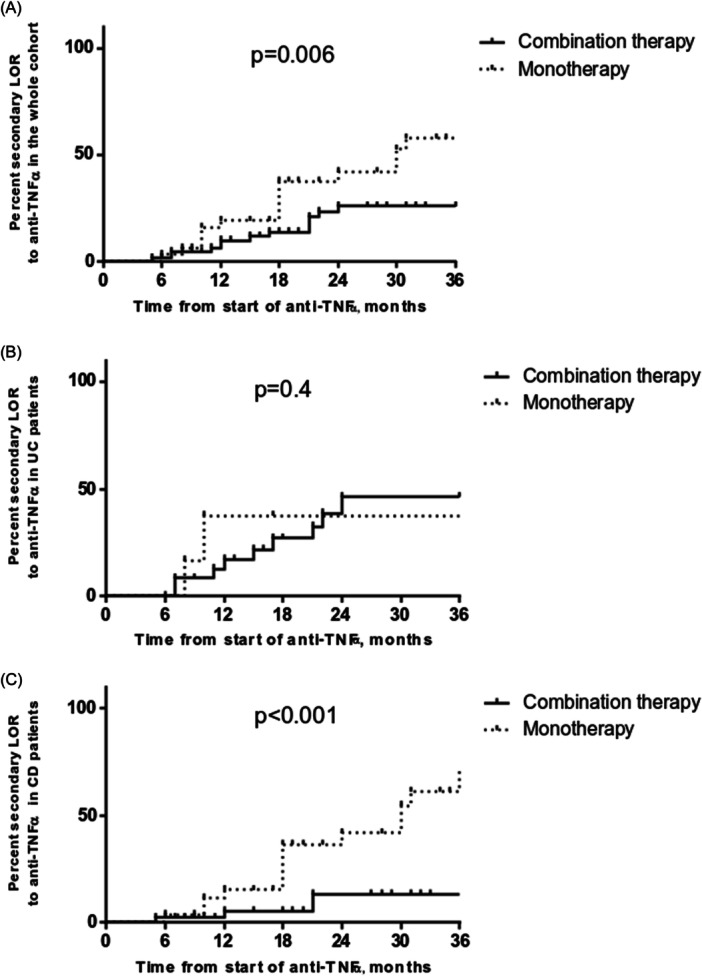
Secondary loss of response to anti TNF‐α agents in pediatric IBD patients treated with combo therapy or monotherapy: (A) Cumulative risk in whole cohort; (B) cumulative risk in UC patients; (C) Cumulative risk in CD patients. CD, Crohn's disease; IBD, inflammatory bowel disease; TNF, tumor necrosis factor; UC, ulcerative colitis.

The median follow‐up after starting biological therapy was 2.6 years (0.5–11.3). Twenty‐three patients out of 80 (28.7%) in combo group showed therapy failure compared with 21/37 (56.8%) children in mono group (*p* = 0.04) (Table 2). Kaplan–Meier analysis demonstrated a significantly increased cumulative probability of anti‐TNF‐α failure in mono group (12 months: 27.7%, 95% confidence interval [CI] 9.1%–50.3%); 24 months: 48.1% [95% CI 29.5%–64.5%]; 36 months: 62.3% [95% CI 45.6%–75.2%]) when compared to combo group (12 months: 21.1% [95% CI 7.7%–38.7%]; 24 months: 35.1% [95% CI 19.8%–50.8%]; 36 months: 35.1% [95% CI 19.8%–50.8%]; log‐rank *p* = 0.003] (Figure 1A). UC patients showed more frequently failure to biological therapy and even earlier than patients with CD (23/43 [53.5%] vs. 21/74 [28.4%], *p* = 0.01; 7 months [1–50] vs. 18 months [2–49], *p* = 0.01). No difference was found when comparing UC children from mono group and combo group (62.5% vs. 51.4%; *p* = 0.7) (Table 2). Therapy failure cumulative probabilities in the UC group were not significantly different when comparing combo group versus mono group [log‐rank *p* = 0.5]) (Figure 1B). CD patients in mono showed significantly increased cumulative probabilities of therapy failure (12 months: 21.1% [95% CI 3.1%–49.8%]; 24 months: 45.7% [95% CI 24.5%–64.8%]; 36 months: 63.8% [95% CI 45%–77.7%]) when compared to CD patients in combo therapy (12 months: 7.4% [95% CI 0%–43.8%]; 24 months: 15.1% [95% CI 0.9%–46.8%]; 36 months: 15.1% [95% CI 0.9%–46.8%]; log‐rank *p* < 0.001) (Figure 1C). Patients on combo therapy showed a lower frequency of secondary LOR when compared to mono group (combo group: 13/80 [16.3%] vs. mono group: 17/37 [45.9%]; *p* = 0.001) (Table 2). Kaplan–Meier analysis confirmed a higher frequency of secondary LOR in children treated with mono versus those undergoing combo therapy (log rank = 0.006) (Figure 2A). When dividing for disease subtype, we did not observe any significant difference in terms of secondary LOR in UC children treated with mono or combo therapy (log‐rank = 0.4) (Figure 2B), while patients affected by CD in mono showed higher cumulative probabilities of secondary LOR (log‐rank < 0.001) (Figure 2C). In both IFX‐ and ADA‐treated patients, combo therapy was associated with a decreased rate of secondary LOR and failure (secondary LOR: IFX‐treated: 18.6% vs. 44%; ADA‐treated: 4.8% vs. 41.7%; *p* = 0.03 and *p* = 0.02, respectively; failure: IFX‐treated: 37.3% vs. 64%; ADA‐treated: 4.8% vs. 41.7%; *p* = 0.03 and *p* = 0.02, respectively). Fourteen out of 117 enrolled children (12%) showed a PNR, without statistically significant difference between two groups (combo group: 10/80 [12.5%] vs. mono group: 4/37 [10.8%]; *p* = 1). Four patients in IFX therapy suspended anti‐TNF‐α due to adverse reactions to biologic therapy (combo group: 1/80 (1.3%) vs. mono group: 3/37 [8.1%]; *p* = 0.09).

**Table 2:** Primary and secondary outcomes in the two study groups.

Characteristics	Combo group (*n* = 80)	Mono group (*n* = 37)	*p*
Overall therapy failure, *n* (%)	23 (28.7%)	21 (56.8%)	0.04
UC	18 (51.4%)	5 (62.5%)	0.7
CD	5 (11.1%)	16 (55.2%)	<0.001
Median time to failure, months (range)	7 (1–24)	15 (1–50)	0.05
UC	6.5 (1–24)	8 (1–50)	0.9
CD	12 (2–21)	18 (5–49)	0.3
PNR, *n* (%)	10 (12.5)	4 (10.8)	1
UC	9 (25.7)	2 (25)	1
CD	1 (2.2)	2 (6.9)	0.6
Median time to PNR, months (range)	4.5 (1–6)	4.5 (1–6)	1
UC	5 (1–6)	2.5 (1–4)	0.3
CD	2 (2–2)	5.5 (5–6)	1
Secondary LOR, *n* (%)	13 (16.3)	17 (45.9)	0.001
UC	9 (25.7)	3 (37.5)	0.7
CD	4 (8.9)	14 (48.3)	<0.001
Median time to LOR, months (range)	15 (5–24)	18 (6–50)	0.3
UC	15 (7–24)	10 (8–50)	1
CD	16.5 (5–21)	18 (6–49)	0.5
Adverse reactions, *n* (%)	1 (1.3)	3 (8.1)	0.09
UC	—	—	
CD	1 (2.2)	3 (10.3)	0.3
Remission at 12 months, *n* (%)	45 (90)	23 (88.5)	1
UC	16 (80)	2 (50)	0.2
CD	29 (96.7)	21 (95.5)	1
Remission at 24 months, *n* (%)	29 (93.5)	15 (100)	1
UC	9 (81.8)	2 (100)	1
CD	20 (100)	13 (100)	1
Remission at 36 months, *n* (%)	11 (91.7)	4 (80)	0.5
UC	3 (100)	2 (100)	—
CD	8 (89.9)	2 (66.7)	0.4

Abbreviations: CD, Crohn's disease; LOR, loss of response; MH, mucosal healing; PNR, primary nonresponse; UC, ulcerative colitis.

#### 3.3.2 TDM

Fifty‐four out of 117 (46.2%) patients in our cohort measured anti‐TNF‐α TL and antibodies during the follow‐up with a total of 130 measurements and a median of 2 (1–6) for each patient. The first TL measurement after anti‐TNF‐α treatment initiation was performed after a median of 6 months (1–36) and 10.5 months (2–36) with a median TL of 3.1 μg/mL (0.02–14.3) and 7.7 μg/mL (0.05–12) for IFX and ADA, respectively. Following induction treatment, adequate TL (≥5 μg/mL for IFX and ≥7.5 μg/mL for ADA) were observed in 24/54 (44.4%) patients. Details of the anti‐TNF‐α treatment and TDM are summarized in Table S2. Thirty‐five patients out of 54 (64.8%) underwent proactive TDM, while 37% (19/54) a reactive approach without differences between the combo group and mono group. Patients undergoing a reactive approach showed more frequently therapy failure when compared to proactive TDM in both groups (combo group: 41.7% vs. 4.3%; *p* = 0.01; mono group: 87.5% vs. 20%; *p* = 0.01). A higher number of patients on combo therapy had adequate TL compared to patients on mono during follow‐up (52.8% vs. 22.2%; *p* = 0.005). Antibodies against anti‐TNF‐α were found in 8/57 (14%) children on IFX therapy, without significant differences between the two groups (combo group: 5/36 [13.9%] vs. mono group: 3/21 [14.3%]; *p* = 1).

#### 3.3.3 Secondary outcomes

Among children continuing anti‐TNF‐α therapy, no statistically significant differences were found between the two groups at 12, 24, and 36 months of follow‐up in term of clinical remission (12 months: combo group: 45/50 [90%] vs. mono group: 23/26 [88.5%]; *p* = 1; 24 months: combo group: 29/31 [93.5%] vs. mono group: 15/15 [100%]; *p* = 1; 36 months: combo group: 11/12 [91.7%] vs. mono group: 4/5 [80%]; *p* = 0.5). At the maximum follow‐up of 36 months, 9/43 (20.9%) patients affected by UC required subtotal colectomy surgery with a median lag‐time from biologic start of 1.9 years (0.7–8.6). No statistically significant differences were found when comparing the two groups (combo group: 6/35 [17.1%] vs. mono group: 3/8 [37.5%]; *p* = 0.3).

#### 3.3.4 Factors associated with anti‐TNF‐α durability

A higher proportion of children continuing anti‐TNF‐α therapy at the end of follow‐up was observed among those receiving combo therapy versus mono therapy (57/80 [71.3%] vs. 16/37 [43.2%]; *p* = 0.004). Patients with CD were more frequently under anti‐TNF‐α therapy when compared with UC children at the end of the follow‐up (53/74 [71.6%] vs. 20/43 [46.5%]; *p* = 0.01). A higher number of children undergoing ADA were continuing anti‐TNF‐α therapy respect to patients under IFX therapy (27/33 [81.8%] vs. 46/84 [54.8%]; *p* = 0.01), while patients managed with proactive rather than reactive TDM (30/33 [90.9%] vs. 8/20 [40%]; *p* < 0.001) were more frequently under anti‐TNF‐α therapy at the end of follow‐up.

In a multivariate regression model, the use of a proactive approach and combo therapy resulted to be independently associated with anti‐TNF‐α durability (odds ratio [OR] = 22.1; 95% CI 3.4–142, *p* = 0.001 and OR = 12.9; 95% CI 1.7–9.7, *p* = 0.01).

## 4 DISCUSSION

To the best of our knowledge, few studies have been conducted comparing combo IM and anti‐TNF‐α therapy versus anti‐TNF‐α monotherapy in pediatric IBD with conflicting results. Our findings demonstrate that the combo therapy seems to be more effective on decreasing overall therapy failure and secondary LOR in children with CD, but not in those affected by UC. Additionally, in line with the pediatric literature, we showed that independently of the association of an IM, a proactive TDM should be preferred over the reactive approach, being associated with a higher probability of reaching adequate TL.

However, it is widely acknowledged that these findings should be interpreted in the context of the study's retrospective design, which is inherently less robust than that of a RCT.

Despite the advent of new biologics and small molecules, anti‐TNF‐α inhibitors remain among the most powerful weapon to induce and maintain remission in IBD. Thus, strategies limiting LOR are still among the IBD top research priorities, particularly in children.^21^ To avoid primary or secondary LOR, the two main strategies are proactive TDM to ensure therapeutic drug concentrations and combo therapy with IM.^19^ The most recent review in pediatric cohort conducted in 2015 by Cozijnsen et al. showed no increased efficacy of combo therapy compared to mono.^22^ Differently, a more recent retrospective study of 315 children with IBD under proactive TDM showed that early IM use significantly prolonged the LOR‐free interval, even though the overall rate of LOR after 60 months did not differ between groups.^23^ The results from our real‐life study reinforce the advantages of combo therapy to maintain anti‐TNF‐α efficacy over time, in both IFX and ADA subgroups. In 2019, Lega and colleagues showed that IFX durability did not differ between young patients on IFX monotherapy based on proactive TDM and patients receiving combo therapy.^4^ The same results were also shown by Kappelman's randomized double‐blind study, which showed that only ADA and MTX combo therapy decreased treatment failure in a cohort of children with CD.^24^ In our cohort, when subdividing patients according to disease subtype, the cumulative probability of LOR to anti‐TNF‐α at 24 months was 13.2% in CD patients treated with combo therapy compared to 41.7% in the group treated with mono. Conversely, we did not find significant differences between the two therapeutic strategies in UC. These findings are consistent with the study from Cheng et al. who demonstrated in 148 pediatric IBD patients that Combo therapy was superior to IFX alone in maintaining remission rates at 1 year (78% vs. 54%) and 2 years (68% vs. 46%) in CD children, but not in the UC cohort.^25^ Why does combo therapy not work in the UC setting? One possible explanation can be referred to the fact that in 65.1% of UC children IFX was used as a rescue therapy for an ASC attack. It has been demonstrated that in the most severe forms of UC, IFX failure could rely on altered pharmacokinetics, including rapid clearance, which leads to reduced drug exposure.^26^ In this setting, it is conceivable that higher levels of anti‐TNF‐α are immediately needed while the combo therapy rather act on the long‐term.

How does combo therapy increase anti‐TNF‐α durability in CD children? It is not clear what the benefit of combo therapy depends on. Previous studies have shown that IM reduces the risk of developing antibodies.^27^ However, prevention of immunogenicity does not completely explain the benefits of combo therapy. Recently, a randomized trial by Kappelman et al. showed that the benefit of combination therapy with low‐dose methotrexate was specifically observed in patients treated with ADA, a biologic known to have lower immunogenicity.^24^ This finding highlights that the efficacy of combination therapy goes beyond immunogenicity control alone. As demonstrated in other studies, the effect of combo therapy may also rely on a direct increase of anti‐TNF levels.^23^, ^24^, ^28^, ^29^, ^30^

During the last decade, the widespread use of TDM has hugely increased our possibilities of understanding anti‐TNF pharmacokinetics. However, up to date it is still unclear whether a proactive optimization of TL may decrease the risk of LOR, with adult RCTs being disappointing.^9^, ^10^ Differently from adults,^31^ we demonstrated that the proactive approach was the most significant factor increasing the long‐term IFX durability in both UC and CD children. These findings reinforce the hypothesis that a proactive approach should be preferred over the reactive. Conversely, our study design does not allow us to prove whether an anti‐TNF‐α monotherapy with proactive TDM might be comparable or even superior to the combo therapy, since mono group patients were not always proactively monitored. Recently, Colman et al. compared IFX proactive TDM mono versus combo therapy in a pediatric cohort of IBD patients demonstrating no differences with respect to IFX clearance and TL between these groups.^32^ On the other hand, it has to be acknowledged that the comparison between proactive and reactive TDM may be biased, as reactive TDM is typically performed in patients with active disease.

We are aware that our study is affected by several limitations. First, the retrospective data analysis may at least be partially responsible for recall bias and does not allow us to completely rule out the possibility of confounding by indication. In addition, our study includes patients over a period of 16 years,with an evolving therapeutic strategy that did not allow to have standardized data, particularly in relation to TL monitoring. More specifically, as reported above, we cannot exclude with certainty that the primary outcome could have been influenced by the fact that not all children in monotherapy were proactively monitored.

## 5 CONCLUSIONS

Due to the considerable amount of time required for the approval of new biological molecules in children, it is necessary to optimize the use of currently available drugs. Our results suggest that the use of combo therapy for CD children and proactive TDM should be considered to reduce the risk of anti‐TNF‐α failure. Further studies with long prospective follow‐up and larger sample size are required to confirm our findings and to provide more specific guidance for clinical application.

## CONFLICT OF INTEREST STATEMENT

Massimo Martinelli received payment/honorarium from Bioprojet, Nestle Health Science and Sandoz. Annamaria Staiano served as a member of advisory board for Abbott and Aboca and received payment/honorarium from Angelini, Bromatech, Janssen Biologics B.V, Nestle, Novalac, PAREXEL International Srl, Sanofi. Erasmo Miele received grant or research support from Nestle Italy and Nutricia Italy, served as a member of advisory board for Abbott and received payment/honorarium from Bioprojet and Ferring. The remaining authors declare no conflict of interest.

## Supporting information

Supplementary Figure 1. Flow diagram of the subjects’ progression through the study.

Supplementary Table 1. Clinical characteristics at the diagnosis of the enrolled children.

Supplementary Table 2. Anti‐TNFα treatment modalities and therapeutic drug monitoring.

## Data Availability

The data that support the findings of this study are available from the authors upon reasonable request and with permission from the Department of Translational Medical Science, University of Naples “Federico II.”
