# Supplementary figures and images for: Anti‐tumor necrosis factor‐α monotherapy versus combo therapy with immunosuppressant in pediatric inflammatory bowel disease: A real‐life study
Source: J Pediatr Gastroenterol Nutr. 2025 Nov 20;82(2):454–64. doi: 10.1002/jpn3.70280 (PMC12864180; doi:10.1002/jpn3.70280)

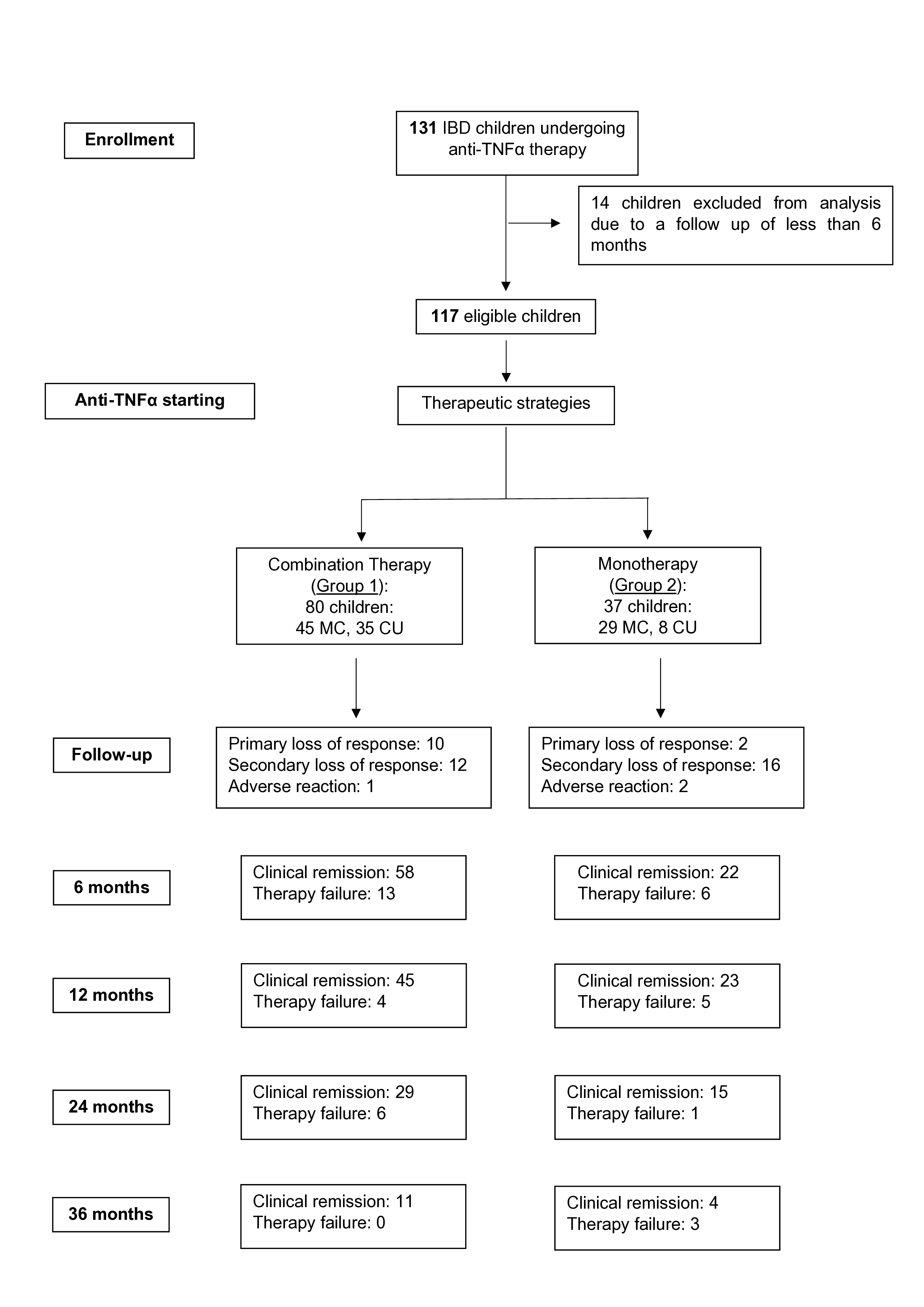

Supplement: Supplementary file 1 — Supplementary Figure 1. Flow diagram of the subjects’ progression through the study. [file JPN3-82-454-s002.tiff]
