# Supplementary material for: Anti‐tumor necrosis factor‐α monotherapy versus combo therapy with immunosuppressant in pediatric inflammatory bowel disease: A real‐life study
Source: J Pediatr Gastroenterol Nutr. 2025 Nov 20;82(2):454–64. doi: 10.1002/jpn3.70280 (PMC12864180; doi:10.1002/jpn3.70280)
Supplement: Supplementary file 2 — Supplementary Table 1. Clinical characteristics at the diagnosis of the enrolled children. [file JPN3-82-454-s001.docx]

**Supplementary Table 1. Clinical characteristics at the diagnosis of the enrolled children ________________________________________________________________________**

**Characteristics CD (n=74) UC (n=43) p**

**______________________________________________________________________________________**

***Median age at diagnosis, yrs (range****)*  11.8 (3.6-16.6) 10.3 (2.1-16.9) 0.01

***Sex (n, %)*** 39 (52.7) 17 (39.5) 0.2

***PUCAI,* n *(range)*** - 50 (5-80)

***PCDAI,* n *(range)*** 25 (0-85) -

**Laboratory values, n *(range)***

ESR (mm/h) 32 (3-92) 33.5 (8-78) 0.9

CRP (mg/dl) 5.1 (0.33-94.2) 0.7 (0.2-28.4) <0.001

Hb (g/dl) 10.8 (7.6-13.9) 11.4 (5.8-14.6) 0.8

Albumin (g/l) 3.5 (2.5-4.4) 4 (2.2-4.7) 0.02

PLT (/mm^3)^ 477x 10^3^ (208-792) 395 x 10^3^ (220-972) 0.1

Fecal Calprotectin (µg/g) 358 (15-4410) 447 (26-2852) 0.4

***Paris classification at the diagnosis (n, %)***

***CD***

Ileum only (L1) 9 (12.3) -

Colon only (L2) 13 (17.8) -

Ileum and colon (L3) 51 (69.9) -

Upper gastrointestinal tract (L4) 15 (20.8) -

B1 50 (67.6) -

B2 6 (8.1)

B3 -

B1+P 18 (24.3%) -

***UC***

Ulcerative Proctitis (E1) - 2 (4.8)

Left-sided colitis (E2) - 5 (11.9)

Extensive colitis (E3) - 3 (7.1)

Pancolitis (E4) - 32 (76.2)

***Induction therapy at the diagnosis (n, %)***

EEN 47 (63.5) -

Steroids 10 (13.5) 31 (72.1) <0.001

5-ASA 4 (5.4) 12 (27.9) 0.001

IFX 2 (2.7) -

EEN +IFX 9 (12.2) -

EEN+ADA 2 (2.7) -

**______________________________________________________________________________________**

CD: Crohn’s disease; EEN: Exclusive Enteral Nutrition; UC: Ulcerative Colitis; IFX: Infliximab; ADA: Adalimumab
