# Supplementary material for: Anti‐tumor necrosis factor‐α monotherapy versus combo therapy with immunosuppressant in pediatric inflammatory bowel disease: A real‐life study
Source: J Pediatr Gastroenterol Nutr. 2025 Nov 20;82(2):454–64. doi: 10.1002/jpn3.70280 (PMC12864180; doi:10.1002/jpn3.70280)
Supplement: Supplementary file 3 — Supplementary Table 2. Anti‐TNFα treatment modalities and therapeutic drug monitoring. [file JPN3-82-454-s003.docx]

**Supplementary Table 2. Anti-TNFα treatment modalities and therapeutic drug monitoring**

**___________________________________________________________________________**

**Characteristics Combo Group (n=80) Mono Group (n=37) p**

**__________________________________________________________________________________________**

**Type of anti-TNF alfa**

**IFX, n (%)** 59 (73.8) 25 (67.6) 0.5

**ADA, n (%)** 21 (26.3) 12 (32.4)

**Proactive approach, n (%)** 23/35 (65.7) 11/19 (57.9) 0.8

**IFX TL (μg/mL) at 1st measurement, median (range)** 3.9 (0.02-14.3) 0.8 (0.04-14.3) 0.07

**IFX TL >5 μg/mL at 1st measurement, n (%)** 11/25 (44) 2/11 (18.2) 0.3

**ADA TL (μg/mL) at 1st measurement, median (range)** 8.37 (0.05-12) 7.1 (4.3-12) 0.5

**ADA TL >7.5 μg/mL at 1st measurement, n (%)** 7/10 (70) 4/8 (50) 0.6

**IFX TL (μg/mL) over follow-up, median (range)** 3.8 (0.04-11.2) 1.5 (0.04-3) 0.03

**IFX TL >5 μg/mL at follow-up, n (%)**  12/25 (48%) 0/9 0.01

**ADA TL (μg/mL) over follow-up, median (range)** 8.7 (0.05-12) 8.4 (4.3-10.9) 0.6

**ADA TL >7.5 μg/mL** at follow-up, n (%) 8/10 (80) 4/7 (57.1) 0.6

**Median TL measurements at follow-up, (range)** 3 (1-5) 2 (1-6) 0.4

**__________________________________________________________________________________________**

IFX: Infliximab; ADA: Adalimumab; TL: trough level
